# An Examination of the Role of CX3CR1 in the Pathobiology of Degenerative Cervical Myelopathy: Evidence from Human and Mouse Tissue

**DOI:** 10.3390/jcm15010082

**Published:** 2025-12-22

**Authors:** Wen Ru Yu, Spyridon K. Karadimas, James Hong, Sarah Sadat, Sydney Brockie, Pia M. Vidal, Tim-Rasmus Kiehl, Noah Poulin, Aikaterini K. Andreopoulou, Joannis K. Kallitsis, Michael G. Fehlings

**Affiliations:** 1 Division of Genetics & Development, Krembil Brain Institute, University Health Network, Toronto, ON M5T 2S8, Canadaspyros.karadimas@gmail.com (S.K.K.); james.hong@uhn.ca (J.H.); sarah.sadat@alumni.utoronto.ca (S.S.);; 2 Laboratory Medicine and Pathobiology, Neuroscience Program, University of Toronto, Toronto, ON M56 1A8, Canada; 3 Department of Chemistry, University of Patras, University Campus Rio, 26504 Patras, Greece; andreopo@upatras.gr (A.K.A.);; 4 Division of Neurosurgery and Spinal Program, Department of Surgery, University of Toronto, Toronto, ON M5T 2S8, Canada

**Keywords:** CX3CR1, microglia, fractalkine, degenerative cervical myelopathy, neuroinflammation

## Abstract

**Background/Objectives:** The molecular cascades involved in the induction and maintenance of neuroinflammation resulting from chronic compression of the cervical spinal cord in the setting of degenerative cervical myelopathy (DCM) have yet to be defined. Here, we determined the role of the fractalkine receptor, CX3CR1, during the neuroinflammatory response in a novel mouse model of DCM and demonstrated the relevance of this mechanism with human DCM tissue. **Methods:** Using our murine DCM model alongside the CX3CR1-knockout mice and a neutralizing antibody of CX3CR1 in wild-type mice, we examined protein, neurobehavioural and immunohistochemical readouts. The animal data were then complemented with immunohistochemical results from human post-mortem spinal cord tissue from individuals with DCM. **Results:** Humans and mice with DCM exhibited an up-regulation of CX3CR1 as well as markers of activated microglia/macrophages in the cervical spinal cord. Knockout and neutralization of CX3CR1 hindered microglia/macrophage activation and accumulation at the site of spinal cord compression. DCM mice exhibited decreased body speed and increased stance phase duration, which mirrors human DCM gait deficits. Strikingly, both CX3CR1 deficiency and CX3CR1 neutralization alleviated these gait deficits in DCM mice. **Conclusions:** Collectively, these data provide strong evidence that CX3CR1 plays a critical role in the secondary injury of neural structures in the setting of DCM. Further, targeting of CX3CR1 represents a promising therapeutic strategy to enhance neurological outcomes in DCM.

## 1. Introduction

Degenerative cervical myelopathy (DCM) is the most prevalent cause of spinal cord dysfunction in adults worldwide, resulting from progressive mechanical compression of the cervical spinal cord [1]. This chronic compression arises from a variety of degenerative changes such as intervertebral disk herniation, osteophyte formation, and ossification of spinal ligaments [2]. Unlike acute spinal cord injury (SCI), which typically involves hemorrhage and a rapid onset of necrosis, DCM is marked by a more insidious course. Patients often develop a gradual worsening of motor function, sensory deficits, and gait impairment due to ongoing mechanical stress on neural structures. Current treatment options focus almost exclusively on surgical decompression, which may halt disease progression but often leaves patients with significant residual neurological deficits [3].

Although the precise molecular mechanisms underlying DCM remain incompletely understood, a key emerging feature is that chronic mechanical compression triggers persistent neuroinflammation in the spinal cord. Previous studies have identified increased infiltration and activation of microglia/macrophages in DCM lesions, mirroring aspects of inflammatory processes observed in other chronic neurodegenerative disorders [4]. However, microglial reactions in DCM may differ from acute CNS trauma such as stroke or SCI, where the inflammatory cascade is initiated by a sudden mechanical or ischemic insult. In contrast, prolonged spinal cord compression in DCM appears to drive sustained inflammation that can exacerbate ongoing tissue damage [2]. Investigating these pathways is essential to identify novel therapeutic targets that could complement surgical decompression and mitigate long-term neurological decline.

Fractalkine (CX3CL1) and its receptor CX3CR1 have recently emerged as important modulators of neuroinflammation and neuron–glia communication [5,6,7]. Under homeostatic conditions, membrane-bound fractalkine expressed by neurons supports normal microglial surveillance. However, during chronic or repetitive injury, the soluble form of fractalkine can recruit additional immune cells and activate microglia, potentially leading to secondary damage. While CX3CR1 is classically recognized on microglia in the CNS, it is also expressed on circulating monocytes/macrophages [8] that can infiltrate the spinal cord after chronic compression. Although CX3CL1–CX3CR1 signaling has been studied in models of SCI and other neurological diseases, its specific role in the pathobiology of DCM remains unclear. CX3CR1 is a seven-transmembrane G protein-coupled receptor that is primarily expressed on the cell surface of microglia and infiltrating macrophages in the CNS, though it can also be found on certain neuronal populations [9,10]. The receptor exists in multiple glycosylated isoforms, typically detected at ~50 and ~55 kDa by Western blotting, which represent differential post-translational modifications. While both isoforms are capable of binding fractalkine and initiating intracellular signaling cascades, the functional significance of differential glycosylation in the context of CNS injury remains to be fully elucidated. Here, we evaluate whether therapeutic modulation of CX3CR1 can provide neuroprotection and improve neurological outcomes in a mouse model of DCM, and we corroborate these findings using human postmortem spinal cord tissue.

## 2. Materials and Methods

### 2.1. Human DCM Spinal Cords and Immunohistochemistry

In this study, spinal cord sections derived from 7 autopsy DCM cases and 7 uninjured control cases were obtained. Sections were provided by the pathology department of the University Health Network (UHN), Toronto, Canada, with approval from the UHN Research and Ethics Board. Table 1 [4] summarizes the relevant demographic and clinical data for each donor, including age, sex, approximate time living with DCM symptoms, spinal level of compression, documented degenerative changes, and cause of death. Ages ranged from 61 to 89 years in the DCM group, with both male and female donors represented. In general, the duration of myelopathic symptoms ranged from several months to over a decade. Most individuals had undergone surgical decompression of the cervical spine shortly before death; causes of death were typically related to cardiac events, respiratory complications, or other non-neurological factors. The control group (ages 48 to 81 years) consisted of donors who had no documented history or pathology of cervical spondylotic myelopathy or other spinal cord disease. In these control cases, histological examination revealed normal spinal cord cytoarchitecture with intact gray–white matter boundaries, minimal microglial activation, and no evidence of demyelination or neuronal loss. Microglia in control tissue displayed ramified morphology characteristic of the surveilling phenotype. In these individuals, the cause of death was similarly varied, predominantly involving cardiac or pulmonary events. Importantly, we excluded any donors with gross macroscopic or histopathological abnormalities of the spinal cord.

Paraffin spinal cord sections of 5 μm thickness were initially deparaffinized in xylene and dehydrated through a series of graded ethyl alcohols. Then the sections were put in a microwave with 10 mM citrate buffer pH 6.0 for 10 min and rinsed three times for 5 min in PBS. Endogenous peroxidase was inactivated by treatment with 0.3% H_2_O_2_ in PBS for 30 min. Sections were blocked with 1% BSA and 5% nonfat milk with 0.3% Triton X-100 for 1 h. Slides were incubated with anti-galectin-3, which is involved in cell differentiation, inflammation and fibrogenesis (1:300, Abcam AB53082, Cambridge, MA, USA), and anti-CD163, which is highly expressed in activated macrophages (1:200, Leica Biosystems CD163-L-CE, Wetzlar, Germany) in blocking solution (5% milk, 1% BSA and 0.3% Triton X-100 in PBS) at 4 °C overnight. After washing in 0.1 M PBS, sections were incubated with biotinylated goat anti-mouse (1:200, Vector Laboratories BA-9200-1.5, Burlingame, CA, USA) or goat anti-rabbit antibody (1:200, Vector Laboratories CA, USA BA-1000-1.5) or fluorescent Alexa Fluor 488 goat anti-mouse secondary antibody (1:200; Sigma-Aldrich AP124JA4, St. Louis, MO, USA) in blocking solution for 1 h at room temperature. Following incubation with biotinylated secondary antibody, avidin–biotin complex (ABC; VECTASTAIN^®^ ABC Reagent, Vector Laboratories PK-4000, Burlingame, CA, USA, as described in kit instructions) and diaminobenzidine (DAB; DAB peroxidase) substrate kit (Vector Laboratories SKU: SK-4100, Waltham, MA, USA) was used for visualization of sliced reaction products. DAPI (1:1000, Invitrogen D3571, Waltham, MA, USA) was used as a nuclear counterstain in fluorescence microscopy. For negative controls, primary antibodies were omitted.

Additional immunostaining of CX3CR1 and Iba1, a marker for microglia/macrophages in human DCM and control cases, was conducted by performing heat-mediated antigen retrieval using citrate buffer (pH 6) followed by blocking slides in TBS + 0.1% Triton X-100 with 5% NGS (normal goat serum) for 1 h. Primary antibodies (Abcam ab8020 Cambridge, MA, USA; CX3CR1, Synaptic Systems 234004 Iba1, Gottingen, Germany) in TBS + 0.1% Triton X-100 and 2.5% NGS were incubated overnight (both at 1:100). Sections were rinsed 3 times with TBS and secondary antibodies (Alexa Fluor 568 Goat Anti-Guinea pig, Invitrogen, A11075, Waltham, MA, USA; and Alexa Fluor 647 Goat Anti-Rabbit, Invitrogen, A21244, Waltham, MA, USA) were added at 1:200 in TBS + 0.1% Triton X-100 (Sigma-Aldrich, T9284, St. Louis, MO, USA) and 2.5% NGS (Vector Laboratories, S-1000, Burlingame, CA, USA) for two hours at room temperature. Slides were washed 3 times in TBS and mounted in Mowiol (Millipore Sigma, 0713-100G, Burlington, MA, USA). Confocal imaging was performed on a Nikon Ti Eclipse (Melville, NY, USA) at 20×.

### 2.2. DCM Mouse Model

All animal protocols were approved by the UHN Research Ethics Board. In this study, we used a mouse model of DCM developed by our laboratory, which facilitated studying DCM in genetically modified mice [11]. In this model, we adapted the microsurgical technique used in the development of our rat DCM model that has been demonstrated to reproduce neurological deficits and key neuropathological features, including gray and white matter loss, astrogliosis, and degeneration of the corticospinal tract that are observed in the human condition [12]. Specifically, we used an engineered aromatic polyether copolymer that was solution casted in thin films and treated in order to increase its porosity in order to promote bone deposition after implantation [13,14]. In brief, a C2-T1 midline incision was made and superficial as well as paravertebral muscles were retracted following the anatomical order. Following C4–5, C5–6 and C6–7 resection of the ligamentum flavum, mild injuries on the periosteal under-surface of C5 and C6 laminae were generated using a microhook. The compression material was then implanted underneath the C5 and C6 laminae. Sham operations consisted of implantation and then removal of the material 30 s post-implantation. A subcutaneous buprenorphine injection (0.1 mg/kg) was given to reduce postoperative pain, and a saline injection (0.1 mL/kg) was given for dehydration. Buprenorphine injections were continued twice a day for 2 days post-op. All animals underwent a thorough examination 24 h post-surgery to detect any neurological deficits that could arise from potential harm to the spinal cord or roots during the operation. If any signs of deficits were observed, the respective animal was excluded from the study. The experimental interventions and outcomes assessments were undertaken using a randomized, blinded protocol. Animals were sacrificed at 5 and 10 weeks post-DCM based on both preliminary data and prior research indicating that these intervals effectively capture distinct phases of disease progression in rodent models of chronic spinal cord compression [12]. At approximately 5 weeks, mild-to-moderate neurological deficits typically manifest, accompanied by early histopathological changes, whereas by 10 weeks, the degenerative process is more advanced, allowing detection of significant alterations in microglial activation, tissue integrity, and motor function. These two time points together enabled us to examine both initial and later-stage responses to chronic compression and evaluate whether any beneficial interventions persist or evolve over time. The overall experimental design and timeline are illustrated in Supplementary Figure S2.

### 2.3. CX3CR1-Knockout Mice

Adult female B6.129P-*Cx3cr1^tm1Litt/J^* (CX3CR1-knockout, *Cx3cr1^−/−^*) mice and their littermate controls (wild-type, WT) were purchased from Taconic (New York, NY, USA). All surgeries were conducted in female C57BL/6 mice aged 10–12 weeks. This age range was chosen to model adult animals while controlling for potential age-related changes in baseline inflammation [15]. They were randomly divided into the following groups (n = 30/group and 15 for each time point): (1) WT sham; (2) *Cx3cr1^−/−^* sham; (3) WT DCM and (4) *Cx3cr1^−/−^* DCM group. Animals were sacrificed at 5 weeks and 10 weeks post-surgical implantation of the material (labeled DCM5 and DCM10, respectively). Heterozygous CX3CR1 mice (*Cx3cr1^+/−^)* were not assessed in the current study. It is possible that heterozygotes show partial attenuation of CX3CR1 expression and intermediate phenotypes, which could be explored in future studies [16].

Only female mice were used in this study. This choice was motivated by documented challenges in managing bladder dysfunction and related urogenital complications in male rodents following spinal cord injury [17]. Consequently, using female mice minimized potential confounding variables related to urinary retention and distress. We acknowledge that our findings may therefore be female-specific and recommend future investigations to explore potential sex-based differences.

### 2.4. Blocking CX3CR1 in DCM Mice

Three weeks after material implantation, when the initial myelopathic neurobehavioural features begin to manifest, an intraperitoneal dose of CX3CR1 antibody (Ab) (rabbit anti-human CX3CR1, Torrey Pines Biolabs TP501, Houston, TX, USA) in saline (20 mg/kg) was given twice per week to WT DCM mice (n = 12) for 7 weeks. The rabbit anti-human CX3CR1 antibody has been previously validated for neutralization studies in mouse models [18,19,20]. While the manufacturer’s datasheet specifies reactivity with human and rat CX3CR1, cross-reactivity with mouse CX3CR1 has been demonstrated in functional studies. The neutralizing antibody binds to CX3CR1 on the cell surface, preventing fractalkine binding and subsequent receptor activation. This mechanism reduces functional CX3CR1 signaling rather than depleting total protein levels, though prolonged antibody binding can lead to receptor internalization and degradation. Regarding CNS penetration of systemically administered antibodies, chronic spinal cord compression in DCM is known to compromise blood–spinal cord barrier integrity, as we and others have previously demonstrated [11,21,22]. This barrier disruption facilitates entry of peripherally administered therapeutic antibodies into the CNS parenchyma. Additionally, CX3CR1 is expressed on circulating monocytes and perivascular macrophages, such that systemic antibody administration can modulate both peripheral and CNS-infiltrating immune cell populations. Controls (n = 12) received vehicle administration (saline). A sham operated group (n = 12) was also included. The animals in all experimental groups were sacrificed at 10 weeks post-DCM (DCM10).

### 2.5. Gait Analysis

The locomotor pattern of each group was evaluated using the CatWalk system (Noldus Information Technology, Wageningen, Netherlands) [12,23], a technique that is well established in our laboratory [12,24,25]. All CatWalk testing was performed between 9 AM and 12 PM under normal light cycle conditions to minimize diurnal variability [23]. An investigator blinded to experimental groups labeled each footprint.

### 2.6. Mouse DCM Spinal Cords and Immunohistochemistry

Mice (n = 3/group) were transcardially perfused with PBS and 4% paraformaldehyde (PFA) solution. 8 mm spinal cord samples centered at the compressed site were dissected and embedded in M1 embedding medium. Transverse sections of 14 μm were cut and blocked in a blocking solution (5% milk, 1% BSA and 0.3% Triton X-100 in PBS) for 1 h. The following primary antibodies in a blocking solution were incubated at 4 °C overnight: Galectin-3 (1:70, R&D systems. Inc. AF11197, Minneapolis, MN, USA); CD11b (1:80, Millipore MABF366, Burlington, MA, USA) overnight at 4 °C. Slides were incubated with fluorescent Alexa 594 or 488 goat anti-mouse, anti-rabbit, or anti-rat secondary antibodies (1:300, Sigma-Aldrich, A-11005, A-11008, A-11006, respectively, St. Louis, MO, USA) for 1 h. DAPI for nuclear staining (1:1000, Invitrogen D3571, Waltham, MA, USA) was used as a general cytological stain in fluorescein-stained sections. Stain specificity was determined by omitting the primary antibody.

### 2.7. Western Blotting

Mice were perfused transcardially with 1xPBS under deep anesthesia with isoflurane pentobarbital. A 0.8 cm length of each cervical spinal cord, centered at the compression epicenter, was harvested, and then individually homogenized in a RIPA buffer (Thermo Fisher Scientific 89901, Ottawa, ON, Canada) at 4 °C. Equal amounts of protein (20–50 µg per lane, depending on the abundance of the target protein) were resolved on 12% or 10% SDS-polyacrylamide gels at 200 V and transferred to nitrocellulose membranes. For each individual blot, the same amount of total protein was loaded across all lanes to ensure accurate comparative quantification. In a subset of assays where n = 2 was used (e.g., galectin-3 or Bcl-xL Western blots), tissue availability limited sample numbers. These assays were included because band intensity patterns were highly consistent across replicates, but statistical interpretations were made cautiously.

Membranes were blocked with 5% non-fat milk for 1 h and incubated with primary antibodies including: (1) Iba1 antibody (1:400, Wako Chemicals USA, Inc. 019-19741, Richmond, VA, USA); (2) galectin-3 (1:1000, Abcam AB53082, Cambridge, MA, USA); (3) β-actin (1:2000, Cell Signaling Technology 5125S); (4) CX3CR1 (1:400, Abcam AB8021, Cambridge, MA, USA), and (5) Bcl-xL (1:100, Cell Signaling Technology 2764S, Danvers, MA, USA) at 4 °C overnight. Membranes were incubated in mouse (1:4000, Sigma 12-349, St. Louis, MO, USA) or rabbit (1:2000, Jackson ImmunoResearch 111-035-003, West Grove, PA, USA) secondary antibodies conjugated to horseradish peroxidase. Reaction products were visualized using ECL. Western Blot Detection kit (Amersham Biosciences Inc., Amersham, UK RPN2108) and ImageLab analysis software (version 6.1; Bio-Rad, Hercules, CA, USA) were used to quantify the amount of protein normalized to the loading control (β-actin). All quantification was performed with discrete lane and band selection. Any bands that were smudged, distorted, or otherwise compromised were excluded from analysis. Representative images are shown in figures; full uncropped blots are provided in Supplementary Materials Figure S1.

### 2.8. High-Throughput Luminex Assay

To determine whether CX3CR1 is involved in changes to the expression of inflammatory mediators produced by activated macrophages and microglia under chronic compression, a customized Luminex panel (MD44) was conducted by Eve Technologies (Lethbridge, AB, Canada) to evaluate differences in cytokine and chemokine profiles between the experimental groups.

### 2.9. Cell Quantification

For cell quantification, 2–3 non-overlapping random fields per tissue section were captured at 20× magnification. Each field represented an area of approximately 0.55 mm^2^. Cell counts were averaged across fields for each animal/case. For mouse tissue, n = 3 animals per group with 2–3 sections per animal. For human tissue, n = 6–7 cases per group with 2–3 sections per case. Results are expressed as cell number per field of view. To quantify CX3CR1 and Iba1 numbers in human DCM and control spinal cord tissue (n = 2 controls, n = 3 DCM; tissue availability limited this subset analysis), confocal images were max projected in ImageJ (version 1.53t; National Institutes of health, Bethesda, MD, USA), and thresholding was applied to distinguish positive staining for these markers from background staining prior to quantification of cell numbers.

### 2.10. Statistical Analysis

**Figure 1:**
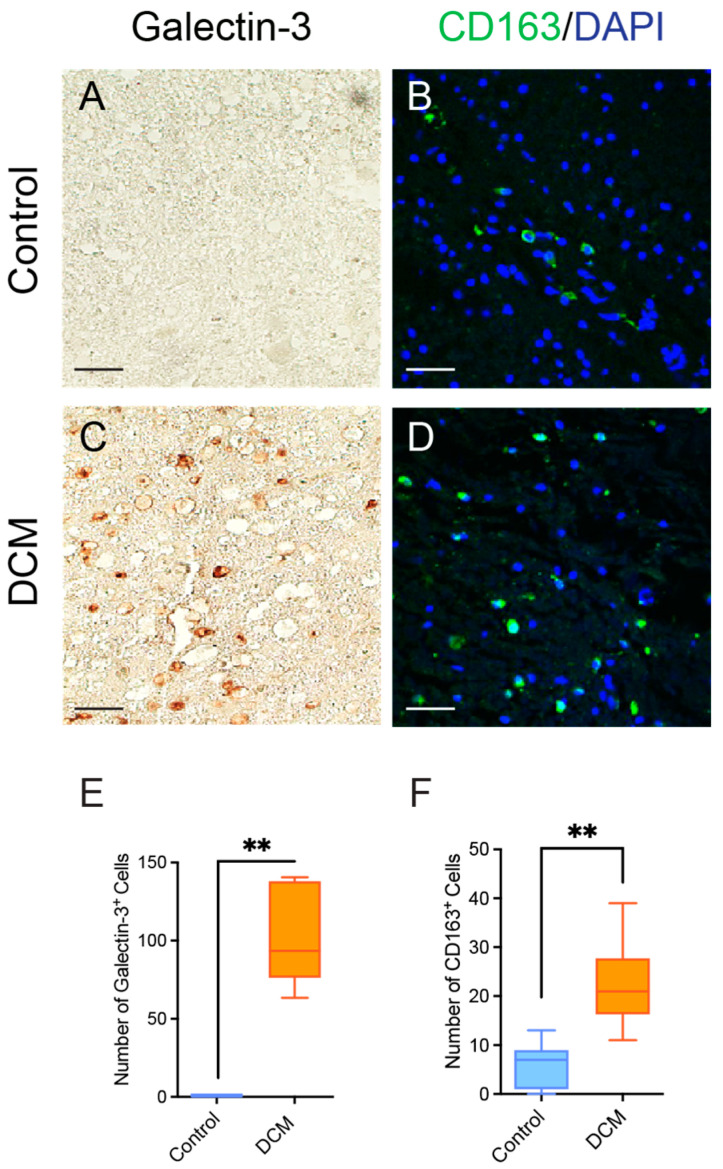
DCM causes upregulation of microglial and macrophage inflammatory markers in humans. Representative cervical spinal cord cross sections of human post-mortem control (**A**,**B**) and DCM cases (**C**,**D**). Samples were stained with galectin-3 in brown (**A**,**C**) and; CD163 in green and DAPI in blue (**B**,**D**). There was a significant increase in the number of galectin-3 + and CD163 + cells (**E**,**F**) in the compressed epicenter field of view of DCM cases compared to controls (** *p* ≤ 0.01, Mann–Whitney U Test). Scale bars indicate 50 µm. Box-and-whisker plots display the median and interquartile range, with individual data points shown. Data points on graphs represent the number of positively stained cells per tissue sample (n = 6/group). DCM, Degenerative Cervical Myelopathy. Representative images are from Control Case #9 (**A**,**D**) and DCM Case #1 (**B**,**E**).

Data analysis was undertaken using Prism 8 software (GraphPad, San Diego, CA, USA). Prior to each analysis, normality was assessed via Shapiro–Wilk tests. For comparisons between two groups, we used Welch’s *t*-test when data were normally distributed (which does not assume equal variances between groups and is more robust than Student’s *t*-test when sample sizes differ) or Mann–Whitney U test when data violated normality assumptions. For comparisons among more than two groups, one-way or two-way analysis of variance (ANOVA) was used for normally distributed data, with Sidak’s post hoc correction for multiple comparisons. Unless otherwise specified, data are expressed as mean ± SEM. For analyses presented as box-and-whisker plots (e.g., Figure 1), data are shown as median with interquartile range and individual data points. Statistical significance is defined as *p* < 0.05. Effect sizes for Welch’s *t*-tests are reported as R^2^ to provide context for the magnitude of observed differences.

## 3. Results

### 3.1. Enhanced Inflammatory Response in Human DCM

To determine DCM-induced neuroinflammatory and anti-inflammatory responses, we performed immunohistochemistry on cervical spinal cord sections from human DCM and uninjured control postmortem tissue. In a previous study, we demonstrated for the first time a key role for inflammation in the pathobiology of DCM in human tissue. Specifically, we had observed significantly increased CD68 and Iba1 expression at the compression epicenter of the spinal cord in human DCM cases [4]. In the present study, we used canonical macrophage neuroinflammatory markers to investigate microglial responses to chronic injury. First, we used galectin-3 to measure classical activation responses of microglia in injured tissue. We observed increased galectin-3^+^ in the human DCM compressed epicenter when compared to control cases (Figure 1A,C, n = 6/group). Although galectin-3 is commonly viewed as a marker of activated microglia and macrophages [26], it can also be expressed by certain astrocytes and neurons. In our images, the majority of galectin-3-positive cells appeared in regions of expected microglial accumulation, suggesting that microglia were likely a primary source. However, we acknowledge that without further co-localization or cell-type-specific morphological confirmation, we cannot fully exclude other cell types that may also express galectin-3.

We then investigated CD163, which has traditionally been thought of as a marker of alternative macrophage activation with anti-inflammatory effects through macrophage contact with endothelial cells in the mediation of immune cell recruitment [27]. Here, we observed significant increases in CD163+ cells (Figure 1D) in DCM compared to only a few CD163+ microglia around vessels in control cases (Figure 1B). The increased presence of CD163+ cells in DCM tissue likely reflects the chronic nature of the injury and the attempt at inflammation resolution, though whether these cells successfully resolve inflammation or represent a failed reparative response requires further investigation. The concurrent upregulation of both galectin-3 (classically pro-inflammatory) and CD163 suggests a mixed activation phenotype that is characteristic of chronic injury states. Future studies should interrogate the phagocytic activity of these cells and employ additional markers (e.g., CD80 for immunostimulatory phenotype, CD206 for resolution phenotype) through multi-label immunofluorescence to more comprehensively characterize functionally distinct subpopulations of macrophages in the context of chronic injury.

### 3.2. Increased CX3CR1 Expression in Human and Murine DCM

To examine whether CX3CR1 contributes to the neuroinflammatory response in DCM, we performed immunohistochemistry on cervical spinal cord sections from human and murine tissue. Two DCM donors (Case #2, 4 days post-anterior cervical disk fusion; Case #4, 4 days post-laminectomy) had recently undergone decompression surgery that could introduce acute inflammation; however, CX3CR1 and microglial marker up-regulation was consistent across all DCM cases, indicating that the findings predominantly reflect chronic compression rather than postoperative change.

**Figure 2:**
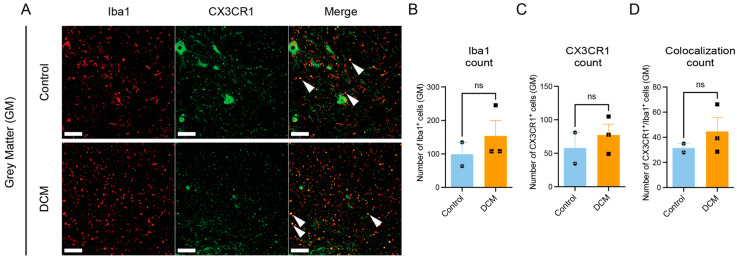

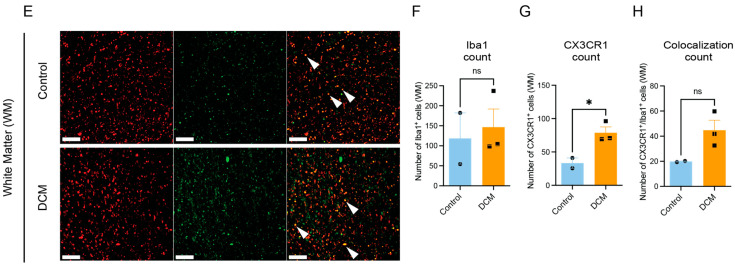
Upregulation of CX3CR1 expression in microglia/macrophages after DCM in humans. Representative immunofluorescence images showing Iba1+ microglia/macrophages (red) and CX3CR1+ cells (green) in gray matter (GM) from control and DCM cases (**A**). Colocalization of CX3CR1 with Iba1+ cells (white arrows) is evident in merged images. Quantification shows no significant difference in Iba1+ cell counts between control and DCM in gray matter (**B**) or CX3CR1+ cell counts in gray matter (**C**), with no significant difference in colocalization counts in gray matter (**D**). In white matter (WM) (**E**), similar staining patterns are observed with colocalized CX3CR1+/Iba1+ cells (white arrows). White matter quantification reveals no significant difference in Iba1+ cell counts (**F**), but a significant increase in CX3CR1+ cells in DCM compared to control (* *p* < 0.05) (**G**), with a trend toward increased colocalization that did not reach statistical significance (ns = not significant, **H**). * *p* < 0.05, Welch’s *t*-test. Scale bars indicate 50 µm. Bars indicate the mean, and error bars represent the SEM (n = 3 DCM, n = 2 controls). Data points represent individual tissue samples, circles = control, and square = DCM. GM, Gray Matter; WM, White Matter; DCM, Degenerative Cervical Myelopathy. Representative images are from Control Case #9 and DCM Case #1.

In human tissue, CX3CR1-positive cells were observed in both gray and white matter, colocalizing with Iba1-positive microglia/macrophages (white arrows in Figure 2A–D). Quantification revealed no significant difference in CX3CR1+ or Iba1+ cell counts within the gray matter between DCM and controls (Figure 2E,F). In contrast, white-matter regions showed a significant increase in CX3CR1+ cells in DCM compared with controls (Figure 2H; Welch’s t(2.86) = 3.90, *p* = 0.033; 95% CI [−83.54, −7.32]; R^2^ = 0.84). Iba1+ counts in the same regions were unchanged (Figure 2G). Although the difference in CX3CR1/Iba1 colocalization did not reach significance, the effect size suggests a biologically meaningful increase in CX3CR1-expressing microglia/macrophages during chronic compression.

**Figure 3:**
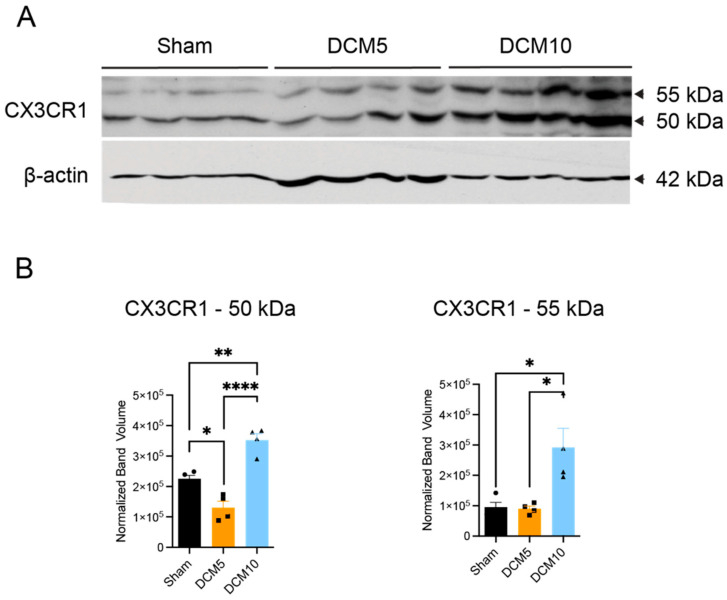
Increased CX3CR1 expression after murine DCM. Western blotting for CX3CR1 shows increases in both the 50 and 55 kDa isoforms of CX3CR1 after DCM (**A**). Densitometric quantification of Western blot shows significant increases in CX3CR1 only after 10 weeks of DCM relative to sham controls (**B**). * *p* < 0.05, ** *p* ≤ 0.01 and **** *p* ≤ 0.0001, one-way ANOVA, post hoc Sidak. Bars indicate the mean, and error bars represent the SEM. Data points on graphs represent normalized band volumes per sample (n = 4/group). DCM, Degenerative Cervical Myelopathy. Note: Some β-actin bands in panel A show lane bleed-through; however, quantification was performed using Image Lab software with careful partitioning of lanes and background exclusion.

In the murine DCM model, Western blotting confirmed increased CX3CR1 protein levels at both the ~50 kDa and ~55 kDa isoforms in WT DCM mice at 10 weeks compared with WT shams (Figure 3A,B; n = 4; one-way ANOVA with Sidak post hoc tests, *p* < 0.01). Expression at 5 weeks remained similar to sham, indicating that CX3CR1 up-regulation emerges progressively with chronic compression. This delayed rise aligns with the slow, insidious course of DCM and implies that early CX3CR1 inhibition—before peak expression—can mitigate downstream neuroinflammatory signaling.

### 3.3. Decreased Microglial/Macrophage Activation and Recruitment in CX3CR1-Deficient DCM Mice

**Figure 4:**
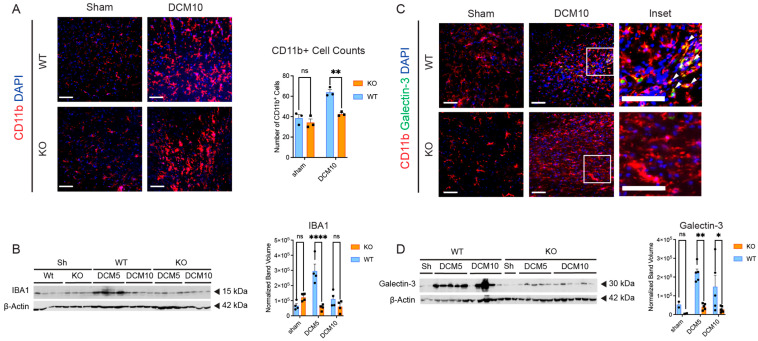
CX3CR1-deficiency attenuates neuroinflammation and microglia/macrophage localization after DCM. (**A**) Immunohistochemistry for CD11b (red) and DAPI (blue) showing reduced CD11b+ cell infiltration in *Cx3cr1^−^/^−^* (KO) DCM mice compared to WT DCM mice at 10 weeks post-DCM. Quantification reveals significantly fewer CD11b+ cells per field in KO DCM compared to WT DCM mice (n = 3/group). (**B**) Western blot analysis of Iba1 (~15 kDa) expression levels across experimental groups with β-actin (~42 kDa) as loading control. Densitometric quantification shows significant differences in Iba1 expression between groups (n = 4/group). (**C**) Dual immunofluorescence for CD11b (red), galectin-3 (green), and DAPI (blue) at 10 weeks post-DCM demonstrating colocalization of CD11b and galectin-3 in activated microglia/macrophages. Insets show high-magnification views of the boxed regions with white arrows indicating colocalized cells. KO DCM mice showed reduced colocalization compared to WT DCM mice. (**D**) Western blot analysis of galectin-3 (~30 kDa) expression with β-actin as loading control. Densitometric quantification demonstrates reduced galectin-3 expression in KO mice compared to WT across time points (n = 2–6/group). ns = not significant, * *p* < 0.05, ** *p* ≤ 0.01, **** *p* < 0.0001; two-way ANOVA with Sidak’s multiple comparisons test. Scale bars indicate 50 μm. Bars indicate the mean, and error bars represent the SEM. Individual data points are shown, in panel A: circle = WT, square = KO; in panels B/C: circle = sham, square = DCM. WT, wild-type; KO, knockout (*Cx3cr1^−^/^−^*); DCM, degenerative cervical myelopathy; DCM5, 5 weeks post-DCM; DCM10, 10 weeks post-DCM; Sh, Sham. Note: Some IBA1, Galectin-3, and β-actin bands in panel B, D show lane bleed-through; however, quantification was performed using Image Lab software with careful partitioning of lanes and background exclusion.

The role of CX3CR1 in neuroinflammation after DCM is not known. To assess this, we performed immunostaining for CD11b and Western blot for Iba1 as markers of microglia/macrophage activation. At 10 weeks post-DCM, CX3CR1-deficient DCM mice had significantly reduced numbers of CD11b-positive microglia/macrophages compared with WT DCM mice (Figure 4A, n = 3/group). Similarly, Western blot analysis revealed that CX3CR1-deficient DCM mice had significantly reduced Iba1 protein levels at both 5 and 10 weeks post-DCM compared with WT DCM mice (Figure 4B, n = 4/group). These findings suggest that the absence of CX3CR1 attenuates the pathological accumulation of microglia/macrophages in the chronically compressed spinal cord. However, whether this reflects decreased infiltration of peripheral macrophages or diminished proliferation of resident microglia remains unclear with the current experiments.

### 3.4. Decreased Inflammatory Responses in CX3CR1-Deficient DCM Mice

To further assess CX3CR1-mediated inflammation after DCM, we performed double immunostaining for CD11b and galectin-3 (Figure 4C), as well as galectin-3 Western blot (Figure 4D) in CX3CR1-deficient DCM mice and WT DCM mice. Galectin-3 is a soluble β-galactoside-binding protein, and is considered to be a neuroinflammatory marker that modulates macrophage and microglial activity [26]. CD11b is another inflammatory marker, and is expressed on the surface of macrophages/monocytes, activated lymphocytes, and microglia in the brain and spinal cord [20].

Dual immunofluorescence revealed minimal galectin-3+/CD11b+ colocalization in both WT sham and CX3CR1-deficient sham mice. However, there were large numbers of galectin-3+/CD11b+ double-positive cells in WT DCM mice at 10 weeks post-injury (Figure 4C). In contrast, CX3CR1-deficient DCM mice showed markedly reduced colocalization of these activation markers. Western blot analysis corroborated these findings, demonstrating that CX3CR1-deficient DCM mice had significantly reduced galectin-3 protein levels at both 5 and 10 weeks post-DCM compared to WT DCM mice (Figure 4D, n = 2–6/group).

### 3.5. Cytokine and Chemokine Activation in CX3CR1-Deficient DCM Mice

**Figure 5:**
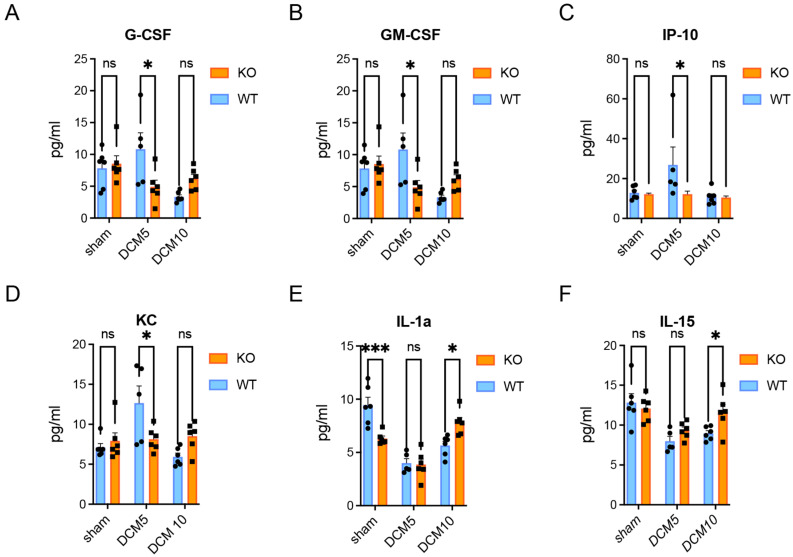
CX3CR1-deficiency changes activation of pro-inflammatory factors in DCM. (**A**–**D**) Luminex results indicated significantly reduced pro-inflammatory protein levels of G-CSF, GM-CSF, IP-10 and KC in the CX3CR1-deficient DCM mice compared to WT DCM mice at 5 weeks post-DCM; (**E**) IL-1α levels showing decreased baseline levels in shams, with increased expression at 10 weeks following DCM in the knockout mice compared to WT; and (**F**) increased anti-inflammatory protein levels of IL-15 in the CX3CR1-deficient DCM mice compared to WT DCM mice at 10 weeks post-DCM. ns = not significant, * *p* < 0.05, *** *p* ≤ 0.001; two-way ANOVA with Sidak’s post hoc test. Bars indicate the mean, and error bars represent the SEM. Data points on graphs represent individual samples (n = 6 for experimental groups, n = 5 for 5 weeks WT DCM group) circle = WT, square = KO. G-CSF, Granulocyte colony-stimulating factor; GM-CSF, Granulocyte-macrophage colony-stimulating factor; IP-10, Interferon γ-induced protein-10; KC, keratinocyte-derived chemokine; DCM, Degenerative Cervical Myelopathy; WT, Wild-type; IL, Interleukin.

**Figure 6:**
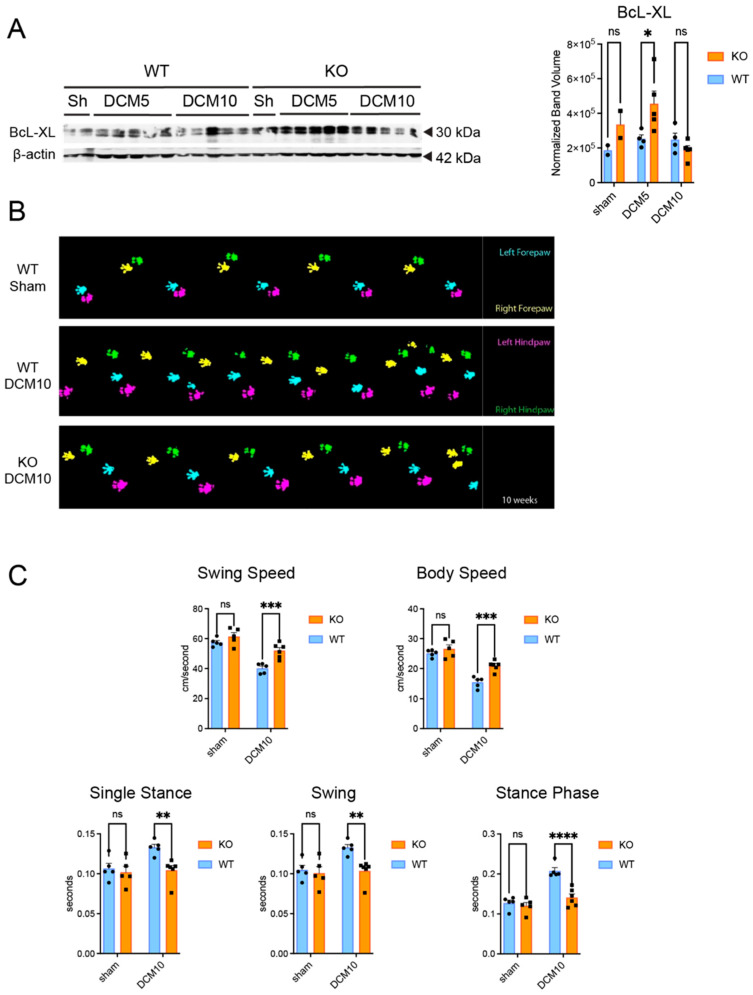
CX3CR1-deficiency increases early expression of anti-apoptotic markers and improves gait after DCM. (**A**) Representative Western blot of Bcl-xL expression in *Cx3cr1^−/−^* and WT DCM mice. Bands were detected at the expected molecular weight of ~30 kDa for Bcl-xL and β-actin was used as a loading control and detected at ~42 kDa. Expected sizes were determined based on antibody datasheets and validated literature. Western blotting demonstrated a significant increase in the level of Bcl-xL between WT and *Cx3cr1^−/−^* animals at 5 weeks post-DCM (n = 2 for Sham, n = 4–5 for DCM); (**B**) murine DCM model recapitulates the gait deficits seen in human DCM, and *Cx3cr1^−/−^* improves gait relative to WT controls; and (**C**) *Cx3cr1^−/−^* improves body and swing speed, mean swing, single stance phase, and stance phase duration relative to WT controls 10 weeks post-DCM. Data points on graphs represent individual samples (n = 5/group, except n = 6 for *Cx3cr1^−/−^* DCM) circle = WT, square = KO. ns = not significant, * *p < 0.05,* ** *p* ≤ 0.01, *** *p* ≤ 0.001, and **** *p* ≤ 0.0001, two-way ANOVA, post hoc Sidak. Bars indicate the mean, and error bars represent the SEM. WT, Wildtype; DCM, Degenerative Cervical Myelopathy. Note: Bands with smudging, poor resolution, or lane bleed-through were excluded from the quantification (panel A; WT DCM5 lane 4, WT DCM10 lane 1; see Table S1 for details).

Luminex array profiling was performed in all experimental groups (n = 6 for all the experimental groups and n = 5 for the 5 weeks WT DCM group). Significantly increased levels of pro-inflammatory factors such as granulocyte colony-stimulating factor (G-CSF), granulocyte macrophage-stimulating factor (GM-CSF), keratinocytes-derived chemokine (KC), and interferon γ-induced protein-10 (IP-10) were detected in WT DCM mice compared to CX3CR1-deficient DCM mice at 5 weeks post-DCM (Figure 5A–D). Strikingly, IL-1α, a known pro-inflammatory cytokine, was significantly increased in sham WT mice compared to sham in CX3CR1-deficient mice (Figure 5E). However, IL-1a levels were significantly increased at 10 weeks in CX3CR1-deficient DCM mice compared to WT DCM mice (Figure 5E). Finally, reduced levels of the IL-15 anti-inflammatory cytokine were found in CX3CR1-deficient DCM mice at 10 weeks post-implantation compared to WT DCM mice (Figure 5F). The Luminex data reveal a complex temporal cytokine profile. Pro-inflammatory cytokines (G-CSF, GM-CSF, IP-10, KC) were significantly elevated at 5 weeks in WT DCM mice compared to CX3CR1-deficient mice, with these differences attenuating by 10 weeks (Figure 5A–D). This pattern suggests that CX3CR1 contributes to early inflammatory amplification in DCM. The IL-1α pattern was particularly interesting: CX3CR1-deficient sham mice showed lower baseline IL-1α than WT shams, but by 10 weeks post-DCM, IL-1α was paradoxically higher in CX3CR1-deficient DCM mice (Figure 5E). This may reflect a compensatory inflammatory response or alternative immune activation pathway in the absence of CX3CR1 signaling. Similarly, IL-15 showed an unexpected pattern with higher levels in WT DCM mice at 10 weeks. While IL-15 has historically been characterized as anti-inflammatory, recent evidence suggests it can have context-dependent pro-inflammatory effects, particularly in chronic neuroinflammation [28]. The anti-apoptotic profile (increased Bcl-xL) observed in CX3CR1-deficient mice at 5 weeks (Figure 6A) may be driven by local tissue responses independent of systemic cytokine levels, highlighting the complexity of CX3CR1’s roles in orchestrating both local cellular survival and broader inflammatory cascades.

### 3.6. CX3CR1 Deficiency Is Associated with an Anti-Apoptotic Shift and Improved Neurological Function in DCM Mice

We next measured the expression of Bcl-xL, an anti-apoptotic marker, to assess whether CX3CR1 deficiency might confer a pro-survival signaling environment. We found a statistically significant increase in Bcl-xL expression in the *Cx3cr1^−/−^* DCM mice compared to WT DCM mice at 5 weeks post-DCM (Figure 6A, n = 2 for Sham and n = 5 for DCM). This finding suggests an upregulation of pro-survival pathways in the absence of CX3CR1.

In addition, we found that the *Cx3cr1^−/−^* DCM mice displayed significantly increased body and swing speed, with corresponding decreases in mean swing and mean stance phase durations, as compared to WT DCM mice (Figure 6C) n = 5/group, except (n = 6) for *Cx3cr1^−/−^* DCM mice. Although these data point to potentially beneficial effects of CX3CR1 deficiency, we did not perform stereological neuron counts or direct apoptosis assays to determine whether such changes translate to neuron-specific protection. Accordingly, further studies are needed to confirm whether increased Bcl-xL expression results in enhanced neuronal survival.

### 3.7. Systemic CX3CR1-Ab Administration in DCM Mice Attenuated Microglia/Macrophage Activation and Increased Neuronal Survival

**Figure 7:**
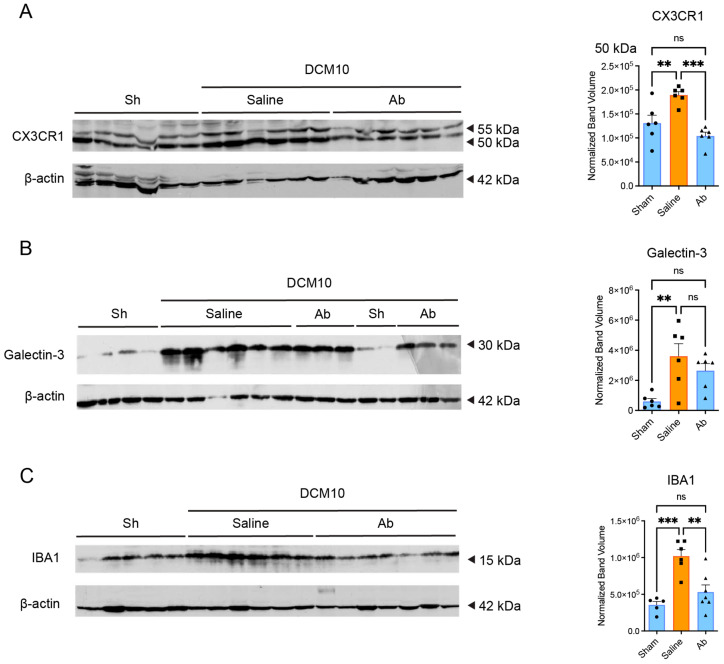
Systemic CX3CR1-Ab administration attenuates microglia/macrophage activation in DCM mice. Western blot analysis revealed a significant reduction in protein levels of CX3CR1 isoforms (~50 and ~55 kDa) (**A**), Galectin-3 (~30 kDa) (**B**), and Iba1 (~15 kDa) (**C**) following systemic administration of a CX3CR1-neutralizing antibody. β-actin was used as a loading control and detected at ~42 kDa. Expected sizes were determined based on antibody datasheets and validated literature. ns = not significant, ** *p* ≤ 0.01 and *** *p* ≤ 0.001, one-way ANOVA, post hoc Sidak. Bars indicate the mean, and error bars represent the SEM. Data points on graphs represent individual samples (n = 5–7/group), circle = sham, square = saline, triangle = CX3CR1-Ab.

To confirm the crucial role of CX3CR1 in mediating chronic microglia/macrophage activation in DCM, and to examine the translational potential of our findings from the deficient animals, purified polyclonal rabbit anti-CX3CR1 (Torrey Pines Biolabs) antibody or saline was injected intraperitoneally in DCM WT mice at a concentration of 20 μg/mL [20,29]. Injections started at 3 weeks post-DCM and were terminated 5 weeks later. Using Western blot at 10 weeks post-DCM, we detected a significant decrease in the levels of the 50 and 55 kDa isoforms of CX3CR1 in DCM mice receiving CX3CR1-Ab (Ab) compared to those which received saline (saline, Figure 7A, n = 6/group), confirming successful attenuation of the CX3CR1 protein.

In agreement with our previous results, we found a statistically significant increase in galectin-3 (Figure 7B, n = 6/group) and Iba1 protein expression (Figure 7C, n = 5–7/group) in the DCM mice that received saline treatment compared to sham mice. However, we detected a significant decrease in galectin-3 and Iba1 protein expression in the CX3CR1-Ab group compared to the saline group. Furthermore, there was no significant difference between galectin-3 and Iba1 expression in sham and CX3CR1-Ab treated animals.

### 3.8. Improved Locomotion After CX3CR1-Ab Treatment

**Figure 8:**
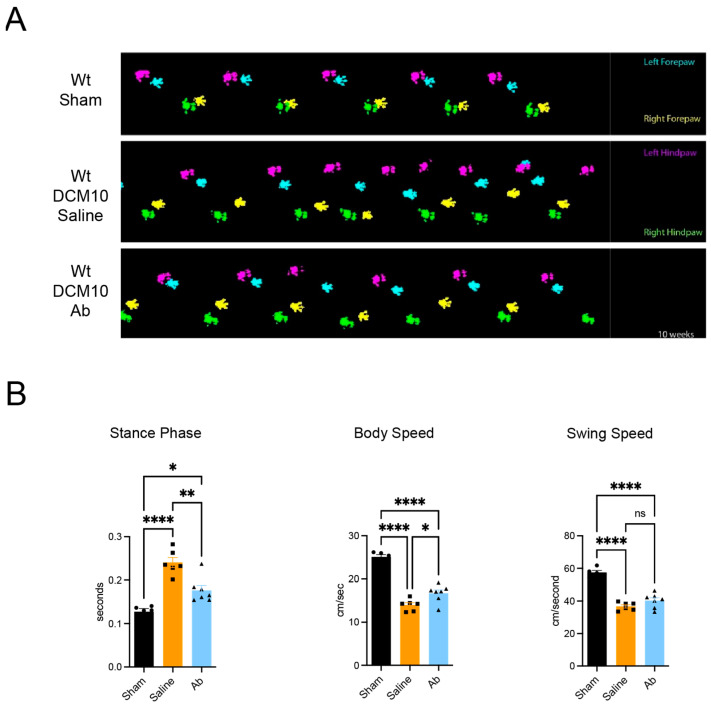
CX3CR1 neutralization abrogates DCM-associated gait deficits. (**A**) CX3CR1 antagonism improves gait pattern relative to saline controls; and (**B**) CX3CR1 antagonism results in a decrease in mean stance phase duration and increase in mean body speed. ns = not significant, * *p* < 0.05, ** *p* ≤ 0.01 and **** *p* ≤ 0.0001, one-way ANOVA, post hoc Sidak. Bars indicate the mean, and error bars represent the SEM (n = 6–7/group). Individual data points are illustrated in the graph, circle = sham, square = saline, triangle = CX3CR1-Ab.

Catwalk gait analysis demonstrated that DCM mice treated with CX3CR1-Ab exhibited improved locomotor gait patterns compared to the control group (Figure 8A). Specifically, post hoc analysis demonstrated significantly decreased mean stance phase duration (n = 6–7/group) and increased body speed (n = 6–7/group) in the CX3CR1-Ab treated group compared to saline controls at 10 weeks post-DCM (Figure 8B).

## 4. Discussion

In this study, we demonstrate for the first time a key role for CX3CR1 in the pathobiology of a murine model of degenerative cervical myelopathy (DCM), supported by parallel findings in human postmortem tissue. CX3CR1 expression was upregulated at the site of chronic spinal cord compression in both species. In the mouse model, both genetic deletion (*Cx3cr1^−/−^*) and antibody-mediated neutralization of CX3CR1 resulted in reduced activation of microglia/macrophages, upregulation of anti-apoptotic signaling, and significant improvements in functional locomotor outcomes.

Although these results are compelling, the mechanistic basis for the reduction in CD11b+ cells in CX3CR1-deficient mice remains unclear. CX3CR1 may influence either the infiltration of peripheral monocyte-derived macrophages or the proliferation of resident microglia. Prior studies in acute spinal cord injury have shown that CX3CR1 modulates chemotaxis [30], supporting this hypothesis. Future studies employing lineage tracing, peripheral immune cell labeling, or in vivo imaging could help disentangle these mechanisms.

DCM is pathophysiologically distinct from acute traumatic spinal cord injury, as it involves chronic mechanical stress without hemorrhagic necrosis [2]. Both animal and human studies have shown that chronic spinal cord compression is associated with increased microglia/macrophage activation [4,31]. These immune cells polarize into either pro-inflammatory or anti-inflammatory phenotypes, influencing disease progression [32,33]. The fractalkine-CX3CR1 axis is known to modulate these immune responses in both injury and disease. Interestingly, the upregulation of CX3CR1 in our human DCM samples closely mirrors the changes observed in the murine model, suggesting that the mechanisms of microglial activation may be conserved across species.

CX3CL1/CX3CR1 signaling exerts context-dependent effects in CNS pathologies. While CX3CR1 deficiency can promote microglial neurotoxicity in some models [16], it improves outcomes in others by reducing inflammation [16]. The divergent effects across injury models may relate to the acute versus chronic nature of the insult, the extent of blood–CNS barrier disruption, and the balance between resident microglia versus infiltrating macrophages. In our DCM model, which features chronic compression without hemorrhagic necrosis, inhibition of CX3CR1 led to reduced microglial activation and better locomotor recovery. These findings align with prior reports in models of multiple sclerosis [16] where CX3CR1 blockade proved beneficial, but contrast with some acute stroke models where CX3CR1 deficiency exacerbated injury [16,34]. This dichotomy likely reflects fundamental differences in injury kinetics: acute injuries may require CX3CR1-mediated microglial surveillance for debris clearance and initial repair, while chronic injuries may suffer from sustained CX3CR1-driven inflammation that becomes maladaptive.

CX3CL1/CX3CR1 signaling exerts context-dependent effects in CNS pathologies. While CX3CR1 deficiency can promote microglial neurotoxicity in some models, it improves outcomes in others by reducing inflammation [16,30,34]. In our study, inhibition of CX3CR1 led to reduced microglial activation and better locomotor recovery. These findings are consistent with prior reports in models of amyotrophic lateral sclerosis and multiple sclerosis [6,7]. We observed improved gait coordination and speed in treated animals, which may represent functionally meaningful recovery. Although these murine gait changes are not directly equivalent to human clinical milestones, they suggest partial restoration of motor function that warrants further exploration.

### Study Limitations and Future Directions

Several limitations should be acknowledged when interpreting our findings. First, although our human data validate the murine observations, the human sample size was small (n = 7 per group) and showed substantial heterogeneity in age, disease duration, compression level, and comorbidities. These factors complicate interpretation of inflammatory markers. Additionally, the DCM group was on average older than controls, though all donors were middle-aged or older, mitigating the potential impact of developmental gene expression differences. Sex differences in DCM prevalence and presentation have been reported, but data are limited. Due to limited tissue availability from autopsy specimens, not all staining protocols could be performed on the complete sample set. We encourage future multi-center autopsy studies or prospective surgical specimen collections with standardized metadata to improve translational relevance. Second, our study used only female mice, which may limit generalizability. This choice was made to reduce complications from urinary retention and prostate-related issues in males, which are exacerbated by spinal cord compression and can lead to severe morbidity. Although this is a widely accepted approach in SCI research, future studies should evaluate sex as a biological variable to identify potential differences in CX3CR1-mediated neuroinflammation. Next, while Bcl-xL upregulation suggests a shift toward a pro-survival environment, we did not perform direct neuron counts or apoptosis assays. It remains unclear whether the observed changes translate to neuron-specific protection. Similarly, although some CX3CR1+ cells appeared morphologically neuronal, we did not perform co-staining with markers such as ChAT due to sample limitations. Prior studies have shown neuronal CX3CR1 expression [10,35] but confirming its presence in DCM-affected spinal neurons will require further dual-labeling approaches. Furthermore, CX3CR1 is not exclusively expressed on microglia, but it is also found on monocyte-derived macrophages and certain neurons. Thus, our approaches (genetic knockout and antibody neutralization) do not provide cell-type specificity. Likewise, we did not investigate downstream signaling pathways in detail. CX3CR1 activation may intersect with PI3K/Akt, MAPK, and JAK/STAT cascades [36,37], but the relative contributions of these pathways in DCM remain to be elucidated. Compensatory mechanisms involving other receptors (e.g., CCR2, TREM2) could also mitigate or obscure the effects of CX3CR1 blockade [38]. Finally, while both genetic and pharmacological inhibition of CX3CR1 improved outcomes in our model, translational application must proceed with caution. Fractalkine signaling is involved in immune surveillance and synaptic pruning [39] and systemic CX3CR1 inhibition could lead to off-target effects, including immune suppression [16] or disrupted CNS homeostasis [40]. More selective, cell-specific, or temporally restricted CX3CR1 inhibition strategies, such as those recently explored by Chen et al. [41], Lund et al. [42], Zhou et al. [43] and Yona et al. [44] may reduce such risks. Additional pharmacokinetic and safety profiling will be necessary to advance this therapeutic strategy.

## 5. Conclusions

Taken together, the findings of this study indicate that the CX3CL1/CX3CR1 signaling pathway plays an important role in mediating the chronic neuroinflammatory response and in the progressive neurodegeneration of DCM. In particular, the activation and recruitment of microglia/macrophages during DCM progression is highlighted. Moreover, data from *Cx3cr1^−/−^* mice using pharmacological antagonization of CX3CL1/CX3CR1 show that disruption of CX3CR1 signaling is associated with functional improvement, reduced inflammation and neuronal damage. This work provides strong evidence that targeting the CX3CR1 signaling pathway represents an attractive approach to attenuate deleterious components of the inflammatory process in the setting of DCM, providing a novel therapeutic target for future studies.

## Figures and Tables

**Table 1:** Summary of the clinical and neurological data in the human DCM and control cases. Case numbers correspond to those referenced in figure legends. DCM, Degenerative Cervical Myelopathy; M/F, Male/Female; C/T, Cervical/Thoracic.

Case	Age	Sex	Level of Compression	Time of Compression	Pathological Findings	Causes of Death
DCM CASES						
1	89	F	C3–8	11-years	Degeneration involved ascending posterior columns; gliosis and C5–8 anterior horn cell loss	Mesenteric infarction with bowel obstruction
2	61	M	C3–6	8-years	Gliosis with cystic cavitation of central gray matter and posterior columns; moderate anterior horn cell loss; degeneration of lateral and posterior columns, dorsal roots and dorsal root entry zone	4 days post anterior decompression and fusion: Acute myocardial infarction
3	75	F	C3–6	>5-years	Focal degenerative lesions in lateral and anterior columns	Pneumonia
4	66	M	C5–T1	3–4 years	Marked anterolateral compression and degeneration and demyelination of corticospinal tracts, posterior and dorsal columns, focal in C5-T1; severe anterior horn cell loss	4 days post laminectomy: acute myocardial infarction
5	73	M	C4–6	4-years	Severe loss of myelinated axons in posterior and lateral columns; loss of motor neurons; spongy degeneration of neuropil	1-day post anterior decompression and fusion, pulmonary edema and cardiac arrest
6	77	M	C5–T2	50-years	Severe anterior horn neuronal loss and gliosis in C7-T1; ascending and descending Wallerian degeneration	6 weeks post anterior decompression and fusion: respiratory compromise and subsequent cardiorespiratory arrest
7	83	M	C3–4	>0.5 months	Demyelination of corticospinal tracts	3 days post anterior decompression and fusion: cardiac arrest
CONTROL CASES						
8	81	F	N/A	N/A	Acute myocardial infarction	
9	72	F	N/A	N/A	Cardiac arrest	
10	66	F	N/A	N/A	Cardiorespiratory arrest	
11	59	M	N/A	N/A	Acute myocardial infarction	
12	52	F	N/A	N/A	Multiple pulmonary emboli	
13	48	M	N/A	N/A	Interstitial lung disease	
14	55	F	N/A	N/A	Renal disease	

## Data Availability

The datasets used and/or analyzed during the current study are available from the corresponding author on reasonable request.

## Supplementary Materials

The following supporting information can be downloaded at: https://www.mdpi.com/article/10.3390/jcm15010082/s1, Figure S1: Full Western blot gels for all figures. Figure S2: Experimental Timeline and Study Design. Supplementary Table S1: Western Blot Band Exclusions.

## Abbreviations

The following abbreviations are used in this manuscript:

Ab	antibody
ABC	avidin–biotin complex
ALS	amyotrophic lateral sclerosis
ANOVA	analysis of variance
CNS	central nervous system
DCM	degenerative cervical myelopathy
DCM5	5 weeks post-DCM
DCM 10	10 weeks post-DCM
G-CSF	granulocyte colony-stimulating factor
GM	gray matter
GM-CSF	granulocyte macrophage-stimulating factor
IP-10	interferon gamma-induced protein-10
KC	keratinocytes-derived chemokine
SCI	spinal cord injury
SEM	standard error of the mean
UHN	University Health Network
WM	white matter
WT	wild-type
