# Supplementary material for: An Examination of the Role of CX3CR1 in the Pathobiology of Degenerative Cervical Myelopathy: Evidence from Human and Mouse Tissue
Source: J Clin Med. 2025 Dec 22;15(1):82. doi: 10.3390/jcm15010082 (PMC12787262; doi:10.3390/jcm15010082)
Supplement: Supplementary file 1 [file jcm-15-00082-s001.zip › jcm-3774023-supplementary.pdf]

Supplemental Figures

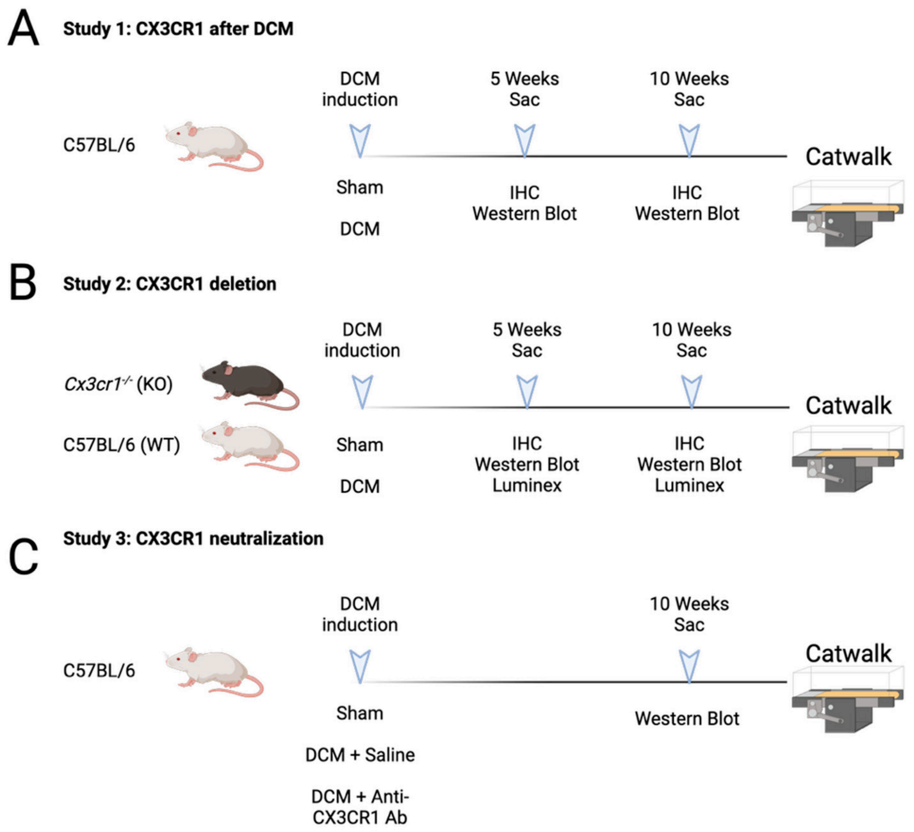

Supplemental Figure S1: Experimental Timeline and Study Design. Schematic representation of the three experimental studies conducted to evaluate CX3CR1's role in degenerative cervical myelopathy (DCM). **(A) Study 1:** Wild-type C57BL/6 mice underwent DCM induction or sham surgery, with sacrifices at 5 and 10 weeks post-surgery for immunohistochemistry (IHC), western blot analysis, and Catwalk gait analysis to characterize baseline DCM pathology and CX3CR1 expression patterns. **(B) Study 2:** CX3CR1 knockout (Cx3cr1-/-) and wild-type (C57BL/6) littermate controls underwent DCM induction or sham surgery, with sacrifices at 5 and 10 weeks post-surgery for IHC, western blot, Luminex cytokine analysis, and Catwalk gait analysis to determine the effects of genetic CX3CR1 deletion. **(C) Study 3:** Wild-type C57BL/6 mice underwent DCM induction or sham surgery, followed by intraperitoneal administration of either CX3CR1-neutralizing antibody or saline control starting at 3 weeks post-DCM. Animals were sacrificed at 10 weeks post-surgery for western blot analysis and Catwalk gait analysis to evaluate therapeutic CX3CR1 neutralization. All groups included both sham and DCM conditions as indicated. DCM, degenerative cervical myelopathy; KO, knockout; WT, wild-type; Sac, sacrifice; IHC, immunohistochemistry.

Supplemental Figure S2: Images of original uncropped Western blots.

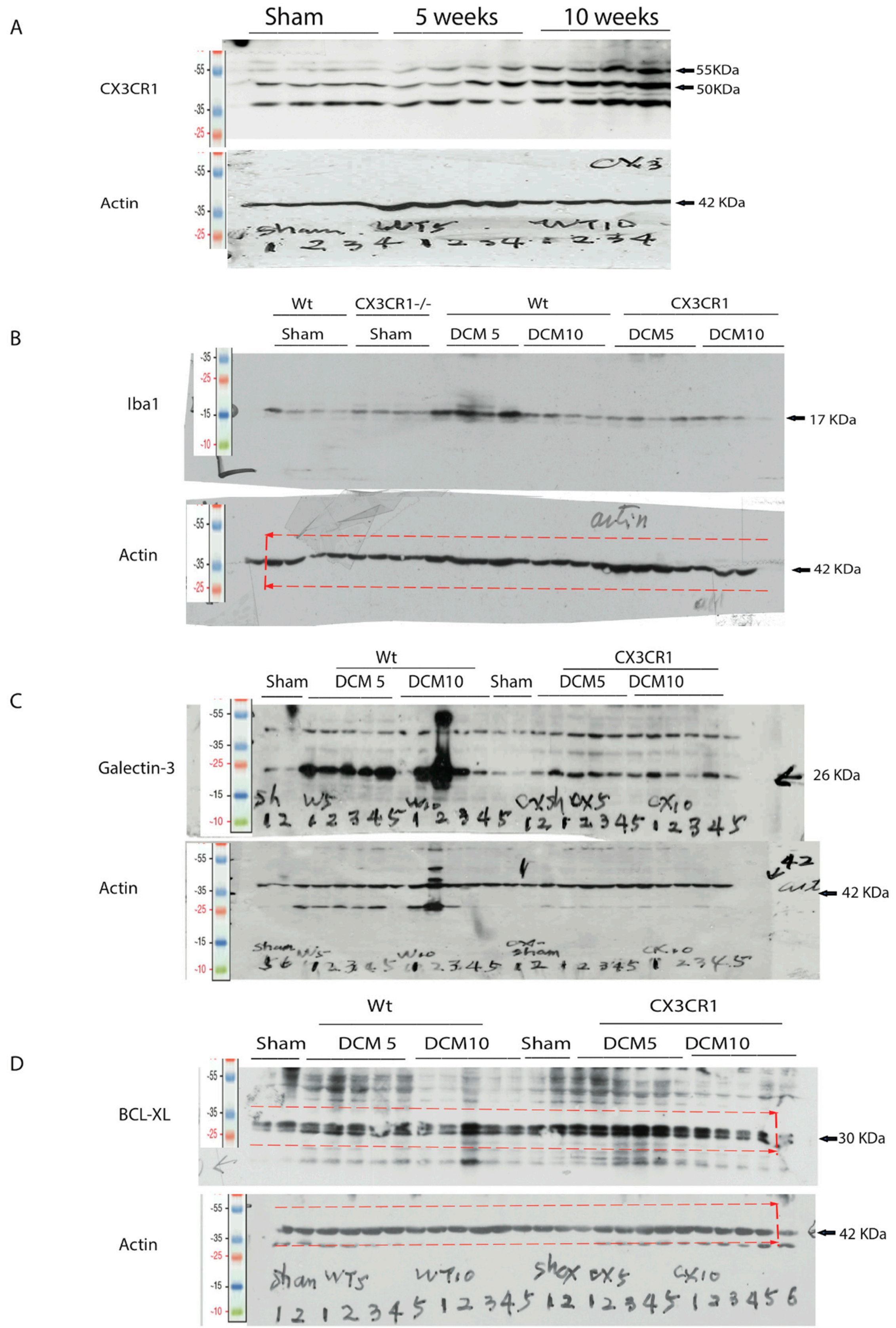

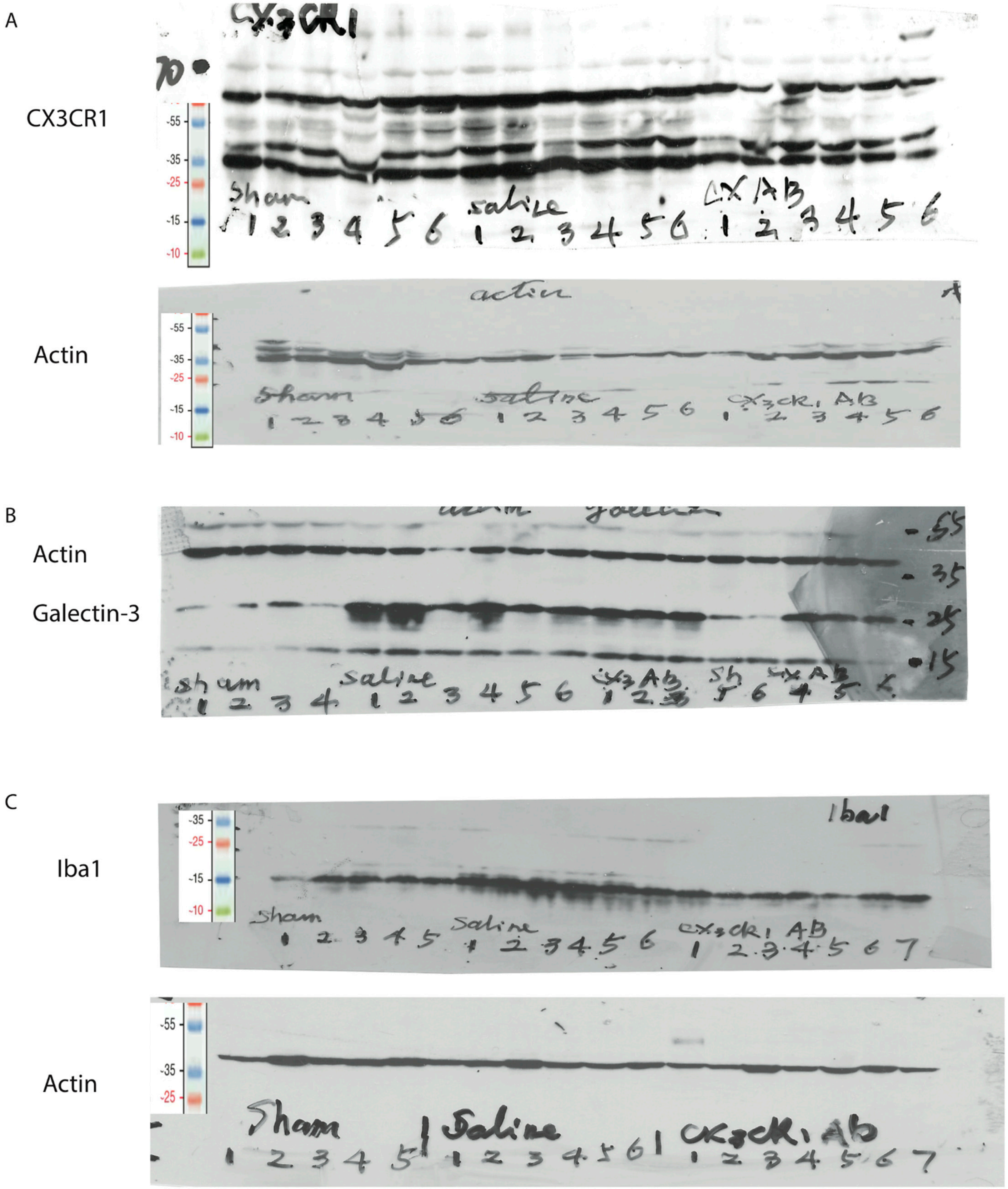
A
CX3CR1
Actin
B
Actin
Galectin-3
C
Iba1
Actin

Supplemental Tables

Supplementary Table S1. Western Blot Band Exclusions. Only bands with clear technical issues affecting quantification accuracy were excluded from analysis. Exclusion criteria included: (1) smudging that prevented accurate densitometric analysis, (2) poor resolution that made band boundaries indistinguishable, and (3) lane bleed-through that contaminated adjacent lanes. All exclusions were determined prior to statistical analysis. Sample sizes (n) reported in figure legends reflect final numbers after exclusions. Abbreviations: WT, wild-type; DCM, degenerative cervical myelopathy; DCM5, 5 weeks post-DCM; DCM10, 10 weeks post-DCM.

Figure	Panel	Protein	Group	Lane	Reason for Exclusion	Final n
**6**	A	BcL-XL	WT DCM5	4	Smudging and poor resolution	4
**6**	A	BcL-XL	WT DCM10	1	Lane bleed-through	4
